# Gender inequality, health expenditure and maternal mortality in sub-Saharan Africa: A secondary data analysis

**DOI:** 10.4102/phcfm.v5i1.471

**Published:** 2013-08-13

**Authors:** Frank Chirowa, Stephen Atwood, Marc Van der Putten

**Affiliations:** 1 Global Health Studies, Faculty of Public Health, Thammasat University, Thailand

## Abstract

**Background:**

This article provided an analysis of gender inequality, health expenditure and its relationship to maternal mortality.

**Objective:**

The objective of this article was to explore gender inequality and its relationship with health expenditure and maternal mortality in sub-Saharan Africa (SSA). A unique analysis was used to correlate the Gender Inequality Index (GII), Health Expenditure and Maternal Mortality Ratio (MMR). The GII captured inequalities across three dimensions – Reproductive health, Women empowerment and Labour force participation between men and women. The GII is a composite index introduced by the UNDP in 2010 and corrects for the disadavanatges of the other gender indices. Although the GII incorporates MMR in its calculation, it should not be taken as a substitute for, but rather as complementary to, the MMR.

**Method:**

An exploratory and descriptive design to a secondary documentary review using quantitative data and qualitative information was used. The article referred to sub-Saharan Africa, but seven countries were purposively selected for an in-depth analysis based on the availability of data. The countries selected were Angola, Botswana, Malawi, Mozambique, South Africa, Zambia and Zimbabwe.

**Results:**

Countries with high gender inequality captured by the gender inequality index were associated with high maternal mortality ratios as compared with countries with lower gender inequality, whilst countries that spend less on health were associated with higher maternal deaths than countries that spend more.

**Conclusion:**

A potential relationship exists between gender inequality, health expenditure, and maternal mortality. Gender inequalities are systematic and occur at the macro, societal and household levels.

## Abstrait

**L’inégalité entre les sexes, les dépenses de santé et la mortalité maternelle en Afrique subsaharienne : Analyse des données secondaires**

**Contexte:**

Cet article propose une analyse de l'inégalité entre les sexes et de sa relation aux dépenses de santé et à la mortalité maternelle.

**Objectif:**

L'objectif de cet article était d’étudier l'inégalité entre les sexes et sa relation aux dépenses de santé et à la mortalité maternelle en Afrique subsaharienne. Une analyse unique a été utilisée afin de corréler l'indice d'inégalité de genre (IIG), les dépenses de santé et le taux de mortalité maternelle (TMM). L'IIG a permis de déceler des inégalité dans trois domaines: la santé reproductive, l'autonomisation des femmes et les différences de participation à la main-d’œuvre entre les hommes et les femmes. L'IIG est un indice composite introduit par le PNUD en 2010 et permet de paliers les inconvénients associés aux autres indices relatifs au genre. Bien que l'IIG intègre le TMM dans son calcul, il convient de ne pas le considérer comme un substitut au TMM, mais plutôt comme un complément de celui-ci.

**Méthode:**

Une étude exploratoire et descriptive associée à une étude documentaire secondaire utilisant des informations quantitatives et qualitatives a été réalisée. L'article fait référence à l'Afrique subsaharienne, mais sept pays ont volontairement été sélectionnés en fonction de la disponibilité des données afin de procéder à une analyse approfondie. Ces pays sont l'Angola, le Botswana, le Malawi, le Mozambique, l'Afrique du Sud, la Zambie et le Zimbabwe.

**Résultats:**

Les pays souffrant d'une forte inégalité entre les sexes, tel qu'indiqué par l'indice d'inégalité de genre, étaient associés à de forts taux de mortalité maternelle, par rapport aux pays présentant une inégalité entre les sexes moindre; de leur côté, les pays consacrant moins d'argent à la santé étaient associés à des taux de mortalité maternelle supérieurs aux taux rencontrés dans les pays où les dépenses de santé étaient supérieures.

**Conclusion:**

Une relation pourrait exister entre inégalité entre les sexes, dépenses de santé et mortalité maternelle. Les inégalités entre les sexes sont systématiques, et se produisent au niveau macro, de la société et du foyer.

## Introduction

The death of a mother is a heavy loss to the family, society and the economy. Women play very important roles including non-paid activities, such as caring for the family and maintaining a healthy home environment. Women's work contributes indirectly to the economic growth of a country and can be likened to investment in health and economic growth.^1^ Further, women also contribute directly to economic growth when they form part of the labour force and are gainfully employed. No cost can substitute ‘mother's care’ for the children and home, which is a heavy loss when women die from avoidable causes. Empirical evidence highlights that the death of a mother harms the overall wellbeing of her children. In Bangladesh, surviving children of a deceased mother are 3–10 times more likely to die prematurely; whilst in Tanzania, children who have lost their mother spend half as much time in school as other children.^2^ In society, women build binding and bridging social capital structures to assist each other, which translate to societal development.^3^

Many women have had their lives cut short because of avoidable deaths during pregnancy. Excess mortality of women in Africa has been socially generated as a result of gender bias in the distribution of health care and other necessities.^4^ Women are dying needless deaths because of their unavoidable reproductive role coupled by gender-biased allocation of health resources. In Afghanistan, the shortage of health services for safe delivery has resulted in pregnancy being likened to a death sentence.^5^

Though the number of maternal deaths has been declining globally, sub-Saharan Africa (SSA) has shown little or no progress. Approximately 358 000 women were dying each year globally as a result of pregnancy according to 2008 estimates^6^ and the latest 2010 estimate is 287 000 deaths.^7^ The global estimates for the year 2008 showed a 34% decline compared with the 2005 estimate (539 358 deaths)^8^, whilst the 2010 estimates show a 20% decline from the 2008 estimates. Despite this decline, SSA still constitutes 58% of all maternal deaths in developing countries. One in every six pregnant women dies in SSA as compared with one in 30 000 in western countries.^9^ These statistics suggest that women's health issues are not being taken seriously in developing countries. Although the situation shows a steady decline, SSA still faces problems that can be attributed to gender inequality and lack of women's empowerment, resulting in troublesome death rates.

The objective of this article is to explore gender inequality and its relationship with health expenditure and maternal mortality in SSA. The analysis used in this article is a unique dimension which tries to correlate the Gender Inequality Index (GII), Health Expenditure and Maternal Mortality Ratio (MMR). The GII will capture inequalities across the three dimensions – Reproductive health, Women empowerment and Labour force participation between men and women. The GII is a composite index introduced by the UNDP in 2010 and corrects for the disadavanatges of the other gender indices^10^. Although the GII incorporates MMR in its calculation, it should not be taken as a substitute for, but rather as complementary to, the MMR. Hence, the relationship between health expenditure and MMR will be analysed separately from the GII in order to establish a potential relationship. The research area of maternal mortality in SSA has received wide attention. However, no study has considered a gender dimension with regard to this problem.

In 1981, the Convention on the Elimination of Discrimination against Women (CEDAW) came into force with efforts to address all forms of discrimination against women. Discrimination in education, employment, decision making, gender-based violence and economic empowerment widens the gender gap and impacts negatively on women's health. The safe motherhood initiative was one of the first actions in response to the CEDAW following a meeting held in Nairobi, Kenya in 1987. The intention of the safe motherhood initiative was to address emergency pregnancy complications in developing countries.^11^ A target of reducing maternal deaths by 50% by the year 2000 was set, but this failed because many developing countries had little access to reproductive health services for women, yet the focus was almost exclusively on skilled attendants at birth and access to emergency obstetric care.^12^ The International Conference on Population Development (ICPD) in 1994 gave prominence to reproductive health and empowering women in the definition of population policy^13^, aiming to reduce the number of adolescent pregnancies by making reproductive health accessible to this group. Women empowerment in reproductive health and control over their body emanated from the ICPD, but gender-biased roles and the lack of financial power kept women as subordinates who are unable to make reproductive health decisions without consulting their husbands.^14^ By 1995, it became clear that inequalities and inadequate expenditure on women's health needs hindered development. These conclusions came up at the World Women's Conference held in Beijing in 1995.^15^ With the intention to improve development for developing nations, heads of state met in New York in September 2000 and drafted the Millennium Development Goals (MDGs). Goal number 5 of the MDGs aims to improve maternal health, setting out two Targets, namely 5a, to reduce maternal mortality ratio by three-quarters by 2015, and 5b, which targets universal access to reproductive health by 2015.^16^ Some aspects of universal access to reproductive health that empowered women as agreed at the ICPD were not fully incorporated in goal number 5. Following strong pressure from developing countries and civic society, access to reproductive health was incorporated in the year 2007 as target 5b (previously discussed) in the MDGs goal number 5.^17^ Gender bias is systematic and occurs at all levels, namely the macro, societal and household levels. The allocation of resources at macro level and household level suffer from gender bias. This adds on to roles we play as a result of our biological differences which put both men and women at risk, affecting their health. The distribution of resources at the household level is based on the contribution to income generation in monetary terms, and as a result, very often more food and resources are made available for men.^18^ Women in SSA constitute 80% of the poor and account for most of the unpaid work.^19^ The nature of work includes taking care of the children at home, preparing the fields, farming, producing vegetables in small gardens, fetching water, collecting firewood, washing clothes, and cooking whilst they get food of fewer calories as compared to men, since women's work is usually not rewarded monetarily.^18^

Women lack the financial resources to allow them to use modern methods of contraception, and lack the ability to negotiate for contraception or safe sex, which increases the chances of dying during pregnancy, because of increased fertility or their contracting HIV.^20^ Governments fail to address the issue of making reproductive health accessible through their responsible ministries. Their expenditures prioritise other issues that do not directly benefit women and save them from needless deaths. High unmet needs in contraceptive usage in Africa signal lack of funds in the field of reproductive health.^7^ Social exclusion and lack of female agency has been the leading cause of poor health states for women. In his paper, ‘*Missing Women’*, Sen highlights the fact that the lack of work outside the home, lack of education and lack of participation in household distribution of resources has resulted in high fertility and high infant mortality rates.^21^ A study by Hobcraft^22^ on the effect of women education on child health found that women with more than seven years of education have on average fewer children in Africa than women with no education. Access to good quality reproductive health care, women education and other interventions may reduce maternal deaths.

**FIGURE 1:**
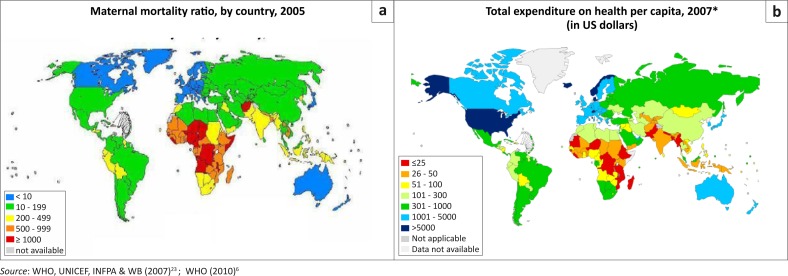
Global comparison of maternal mortality and per capita expenditure: (a) maternal mortality ratio by country, 2005 and (b) Expenditure on health per capital, 2007.

Figure 1 shows the distribution of maternal deaths and health expenditure per capita on two maps. Maternal mortality ratios are higher in SSA (orange and brown colour), where deaths range from 200–1000+ women per 100 000 live births. On the other hand, SSA countries spend less than $100 per individual citizen and the trend is similar for the Asian countries. Australia, Europe and some parts of America have a high expenditure on health as compared to Africa and Asia and their maternal deaths are less than 10 per 100 000 live births. The interpretation that can be drawn from these two maps is that countries that spend more on health are more highly associated with reduced maternal mortality than those that spend less on health. It is not always true that countries that spend more on health are associated with lower maternal deaths, but increased expenditure which is well distributed and associated with more frequent and more intensive use of health services in private and public sectors will have positive health outcomes for women.^24^ Income should be spent equitably on better nutrition, food production capacity, access to affordable reproductive health, transport, water and sanitation, all of which have an impact on women's health. Failure to spend income equitably could manifest as maternal death.

## Design and methods

An exploratory and descriptive design to a secondary documentary review using quantitative data and qualitative information was used for an in-depth analysis of gender inequality captured by the GII, MMR and government health expenditure. Documentary reviews involve the analysis of documents that contain information about the phenomena that the researcher wants to study.^25^ The GII has three dimensions which capture inequality between men and women in a country and is interpreted as a percentage loss in human development as a result of inequality. The three dimensions captured by the GII are the reproductive health, empowerment and labour market components. A descriptive analysis of secondary data was used for the quantitative data whilst content analysis of secondary information was used for the qualitative information. The article focused on SSA, but for in-depth data analysis, Angola, Botswana, Malawi, Mozambique, South Africa, Zambia and Zimbabwe were purposively selected based on the availability of data to compare the relationship between health expenditure, gender inequality and maternal mortality. The quantitative data was obtained from the WHO data base^26^ (maternal mortality ratios and health expenditures), whilst the GII was obtained from the UNDP data base.^27^

## Results

**FIGURE 2:**
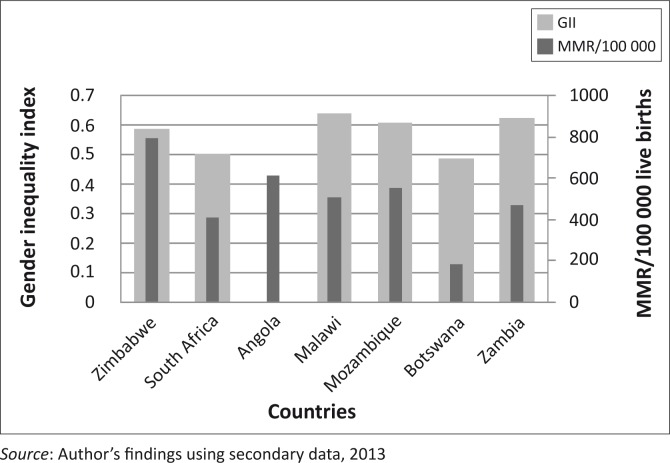
Relationship between Gender Inequality Index and Maternal Mortality.

The GII, which captures disparities between men and women across three dimensions (reproductive health, women empowerment and employment) is shown in Figure 2. Botswana had the lowest GII of slightly below 50%, whilst Malawi had the highest GII of above 60%. No GII data was recorded for Angola. Malawi, Mozambique, Zambia and Zimbabwe had a GII of greater than 50%, representing more than 50% loss in development due to gender inequality, whereas Botswana and South Africa had a GII of below 50%.

Countries with a high GII also had a high MMR as compared with countries with lower GIIs. Malawi, Mozambique, Zambia and Zimbabwe had a higher MMR and GII than Botswana and South Africa. An explanation for this association could be because Botswana and South Africa invested in data for decision-making approaches in order to investigate causes of maternal deaths and identify possible solutions. This enabled them to address health problems and to reduce fertility and maternal mortality rates. Fertility and maternal mortality rates are indicators of the reproductive health component, which constitutes about 73% of the GII in SSA.^19^ Thus efforts to reduce fertility rates and maternal deaths reduce the GII, whilst at the same time reducing the MMR.

Gender inequality and discrimination impede women's access to health, thus limiting their ability to respond to the consequences of ill-health.^28^ This will result in a high GII for such countries and, since some of the inequalities hinder the health-seeking behaviour of women, maternal deaths are likely to increase. The patriarchal system which is prevalent in Africa and SSA in particular feeds in to the high GII. Women have low decision-making power, low education compared with men and own less resources than men. The failure of women to participate in decision making goes beyond the household level to a macro decision-making level. This is reflected in government policies that do not favour women's needs. Lack of access to water and sanitation, reproductive health needs and girls’ education reflect a lack of gender-sensitive national policies, with an end result of high GII and high MMR.

**FIGURE 3:**
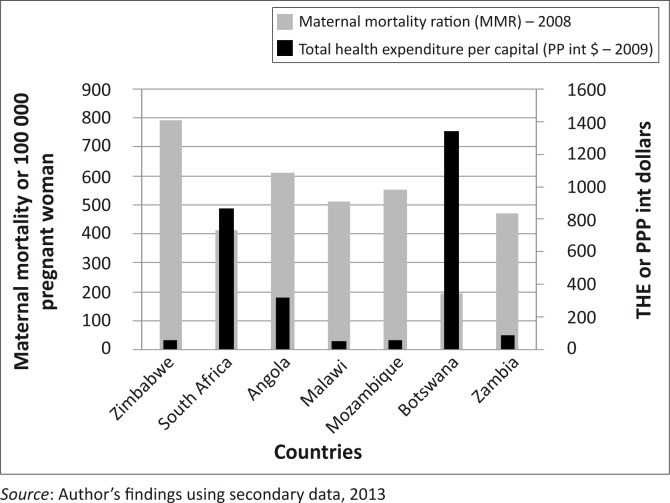
Relationship between Total Health Expenditure (THE) in Purchasing Power Parity (PPP) and Maternal Mortality.

The variable Total Health Expenditure per capita in purchasing power parity terms (THE/PPP) captures preventative care, curative care, nutrition and reproductive health per person. This indicator shows how much a country spends on one person and this figure is adjusted for purchasing power differences using the international dollar to enable the figures to be comparable across countries. In Figure 3, the countries are on the horizontal axis and MMR and THE in $PPP are shown on the two vertical axes.

Botswana and South Africa have the highest health expenditure per capita as compared against Angola, Malawi, Mozambique, Zambia and Zimbabwe. Botswana has the highest expenditure (close to $1400 per person) whilst South Africa spent slightly above $800 per person. As shown in Figure 2, Botswana and South Africa had lower GII and MMR compared with other countries. This relationship seems to go beyond GII and MMR to show that these countries spend more per person compared with the other countries, thus bettering their GII and MMR statistics. Angola is the third best, spending close to $300 per person, whilst Malawi, Zambia and Zimbabwe spend less than $100 per person. Countries that spend more on health had lower maternal deaths than those that spend less. Botswana and South Africa, with the highest expenditure, have lower maternal deaths than Angola, Malawi, Mozambique, Zambia and Zimbabwe. This is a general potential association obtained from this data, but is not always true, as discussed in previous section. Angola is a good example and spends more on health than Malawi, Mozambique and Zambia, but MMR is higher for Angola as compared with the other three countries. This difference might be explained by improper allocation of resources in areas of need, poor governance and misuse of entrusted power for personal reasons other than the country's needs.

## Ethical considerations

Ethical concerns are waived for review studies and the documents incorporated in this study were screened based on their authenticity, credibility and representativeness in covering gender inequality, maternal mortality and health expenditure. Furthermore, secondary data that was used for analysis came from credible sources that have met ethical requirements.

## Discussion and conclusions

The findings indicate a potential relationship between gender inequality as captured by the GII, maternal mortality and health expenditure per capita. The article showed that countries that had higher expenditure on health had lower GII and lower maternal deaths. Lower expenditure and discrimination against women, which were captured by the GII, affect the health of women, with the worst-case scenario being maternal deaths. However, it is acknowledged that the potential relationships were based on the findings of seven purposively selected countries only.

Hypotheses on potential relationships will require further research in order to accept or reject established eventual associations. Further caution needs to be observed in the interpretation of findings since this review was not able to obtain direct expenditures on reproductive health and other factors affecting women's health in the analysis of the gender implication of these expenditures.

It is also acknowledged that important other factors may affect women and contribute to maternal deaths, such as distance to health centres, lack of health insurance, unaffordable user fees, poverty, and lack of commitment by African governments to place maternal mortality onto the policy agenda. All of these factors fall into the category of systematic gender problems, which could be at a household, societal and/or macro level. Increasing financial resources for education, reproductive health, water and sanitation, transport, and increasing access to reproductive health may reduce maternal deaths and improve gender equity. African governments can scale up their efforts to increase enrolments of girls in primary and secondary education, and completion of these educational milestones, in order to improve the girls’ decision making skills, allowing for the likelihood of lowering the pregnancy rate and reducing the risk of maternal deaths. In summary, countries with high GII and MMR had lower expenditure on health as contrasted against countries that spent more on health.
